# Antimicrobial Activity and Genetic Profile of Enteroccoci Isolated from Hoopoes Uropygial Gland

**DOI:** 10.1371/journal.pone.0041843

**Published:** 2012-07-24

**Authors:** Magdalena Ruiz-Rodríguez, Eva Valdivia, Manuel Martín-Vivaldi, Antonio M. Martín-Platero, Manuel Martínez-Bueno, María Méndez, Juan M. Peralta-Sánchez, Juan J. Soler

**Affiliations:** 1 Departamento Ecología Funcional y Evolutiva, Estación Experimental de Zonas Áridas (CSIC), Almería, Spain; 2 Grupo Coevolución, Unidad Asociada al CSIC, Universidad de Granada, Granada, Spain; 3 Departamento Microbiología, Universidad de Granada, Granada, Spain; 4 Departamento Biología Animal, Universidad de Granada, Granada, Spain; 5 Departamento Biología de la Conservación. Estación Biológica de Doñana (CSIC) Sevilla, Spain; Smithsonian Institution National Zoological Park, United States of America

## Abstract

Symbiotic microorganisms may be directly transferred from parents to offspring or acquired from a particular environment that animals may be able to select. If benefits for hosts vary among microbial strains, natural selection may favour hosts holding the most beneficial one. *Enterococci* symbionts living in the hoopoe (*Upupa epops*) uropygial gland are able to synthesise bacteriocins (antimicrobial peptides that inhibit the growth of competitor bacteria). We explored variability in genetic profile (through RAPD-PCR analyses) and antimicrobial properties (by performing antagonistic tests against ten bacterial indicator strains) of the different isolates obtained from the uropygial glands of hoopoe females and nestlings. We found that the genetic profile of bacterial isolates was related to antimicrobial activity, as well as to individual host identity and the nest from which samples were obtained. This association suggest that variation in the inhibitory capacity of *Enterococci* symbionts should be under selection.

## Introduction

Microbial infection is one of the major causes of natural mortality during early life stages, and any trait that reduces bacterial detrimental effects would rapidly be fixed in host populations [1]. A wide variety of animals and plants harbours symbiotic microorganisms that contribute to host defence against parasites [2], mainly through the production of chemical defences. Examples of such symbionts occur in plants [3], invertebrates as marine isopods [4], shrimps and lobsters [5], [6], ants [7] or aphids [8], and vertebrates such as salamanders [9] or birds (reviewed in [10]).

In the case of birds, part of the chemical defences against microbial infections is component of the uropygial gland secretion (UGS hereafter) [11], [12]. In hoopoes (*Upupa epops*), metabolites produced by symbiotic bacteria inhibit bacterial growth [13]. Moreover, the strain *Enterococcus faecalis* MRR 10-3 isolated from the uropygial gland of hoopoes produce at least two bacteriocins (prokaryotic peptides ribosomically synthesized with antimicrobial activity [14]) with a broad antimicrobial spectrum [15]. Bacteriocins could confer protection against potential pathogens to brooding adults and developing nestling hoopoes. The inhibition of bacteriocins in nests of hoopoes increased hatching failures [16], while the bacteriocin MR 10 isolated from hoopoes inhibited growth of keratinolytic bacteria, avoiding feather degradation in lab conditions [17]. Symbiotic bacterial strains are likely vertically transmitted from mother to offspring and, therefore, different individual hosts within a species may harbour different symbiotic bacterial strains with different antibiotic properties [18], which would imply a disparity of potential benefits for hosts [2], [19]. This variation might have an environmental component (as found in bird plumage [20]) if for instance nest environment (including brooding females) determines availability of bacterial strains that will colonize hosts, and a genetic component if strains of similar genetic profiles also have similar antibacterial properties. Determining genetic and antimicrobial profile of symbionts, as well as similarity in genetic profiles and antimicrobial properties of isolates from the same individual and nest, is therefore of prime importance as a first step for understanding coevolutionary processes driving the evolution of possible mutualistic relationships.

In the present work, we studied the variation in antimicrobial activity of genetically characterized enterococci bacterial strains isolated from the uropygial gland of nestling and adult hoopoes. We explored covariation between antimicrobial activity and genetic profiles of the isolates, which were fingerprinted and then clustered in genotypes by using the Random Amplification of Polymorphic DNA (RAPD) analyses. Furthermore, we analysed whether individual hoopoe and nest identities from which the bacteria came from explained antimicrobial properties and genetic profile of isolates. If variation in antimicrobial properties and genetic profile exists, a significant effect of individual and nest identity would suggest the existence of processes favouring particular bacterial strains in particular environments (nests and/or individuals).

## Results

### RAPD Analyses

Samples were divided in 57 RAPD groups. Probability of detecting bacterial colonies of a target RAPD group within the same individual (mean (SE) = 41.89±0.01%) was higher than that expected from a random distribution (mean (SE) = 10.2±0.02) (pair-wise t-test, t = 6.48, df = 37, P<0.0001). Similarly, probability of detecting a target RAPD group in the uropygial secretion of two individuals within the same nest (mean (SE) = 16.29±0.04) was higher than that expected from a random distribution among individuals of different nests (mean (SE) = 6.08±0.02) (pair-wise t-test, t = 3.21, df = 18, P = 0.005). These results suggest an association between genetic profile of bacterial colonies and individual and nest identity where the colony came from.

All analysed 16S rRNA sequences of isolates belonged to the genus *Enterococcus*: *E. faecalis* (N = 27, 45%), *E. faecium* (N = 11, 18.33%), *E. mundtii* (N = 11, 18.33%), *E. casseliflavus* (N = 6, 10%), *E. avium* (N = 2, 3.33%), *E. saccharolyticus* (N = 2, 3.33%), and *E. gallinarum* (N = 1, 1.66%).

### Antimicrobial Activity of Isolates

**Figure 1:**
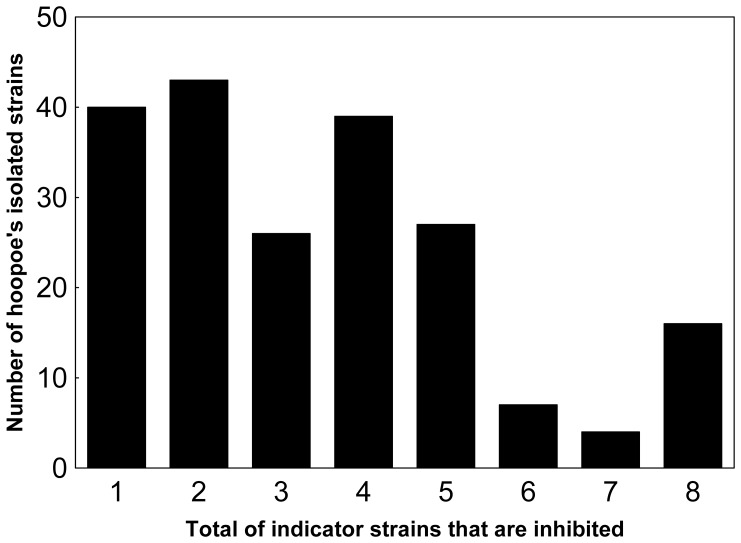
Antimicrobial activity rate. Variation in the number of symbiotic colonies isolated from hoopoe’s uropygial gland secretion that presented antagonistic activity against indicator bacteria.

**Figure 2:**
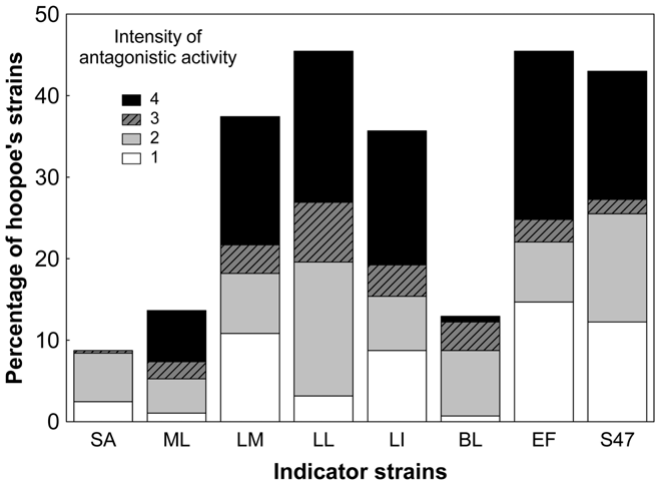
Intensity of antimicrobial activity. Variation in the inhibitory intensity against each indicator strain by symbiotic bacteria isolated from the uropygial gland secretion of hoopoes. (SA: *S. aureus*; ML: *M. luteus*; LM: *L. monocytogenes*; LL: *L. lactis*; LI: *L. innocua*; BL: *B. licheniformis*; EF: *E. faecium*; S47: *E. faecalis*). Intensity of antagonistic activity: 0 (no halo), 1 (ring width <1 mm), 2 (ring width = 1–2 mm), 3 (ring width = 3–4 mm), and 4 (ring width >4 mm.).

Antagonistic activity was found in 202 colonies (70.73%) isolated from hoopoe’s uropygial glands. Those colonies belonged to 55 individuals (95%) from all the 24 nests (100%). Only 3 individual nestlings growing in 2 different nests didn’t show any antagonistic activity against the indicator strains used. In the resulting 57 RAPD groups, antimicrobial activity was detected for 45 of them (78.94%). The number of indicator strains that an isolate was able to inhibit varied from 0 to 8 (Figure 1) with mode and average values of 2 and 2.42 respectively. Moreover, antimicrobial activity and its intensity against different indicators were also very variable (Table 1, Figure 2). The indicator species against which the symbiotic bacteria of hoopoes showed the greatest and the less activity were respectively *L. lactis* and *S. aureus*.

**Table 1:** Inhibition rates (%) for: isolated strains, nests, individuals and RAPD groups against each indicator bacteria.

Indicator bacteria	N° of isolates	N° of nests	N° of individuals	N° of RAPD groups
*Lactococcus lactis*	45.45	95.83	63.79	14.82
*Listeria monocytogenes*	37.41	70.83	51.72	11.97
*Enterococcus faecalis*	43	75	58.62	14.82
*Enterococcus faecium*	45.45	87.5	72.41	18.24
*Listeria innocua*	35.66	75	55.17	13.68
*Staphylococcus aureus*	8.74	12.5	12.06	3.99
*Micrococcus luteus*	13.63	33.33	17.24	7.41
*Bacillus licheniformis*	12.93	33.33	24.13	6.84

### Relationship between Antimicrobial Variation and Genetic Profile

Variation in antimicrobial activity as reflected by the three PCA axes was significantly explained by nest identity (Wilks’ λ = 0.004; F_66, 487.61_ = 39.15; p<0.0001), individual identity (Wilks’ λ = 0.017; F_81, 488.39_ = 17.61; p<0.0001), and by the genetic profile of the isolates (i.e. RAPD group, Wilks’ λ = 0.028; F_123, 489.27_ = 9.14, p<0.0001). Univariate results showed that these effects were highly significant for all dependent variables (PCA axes) analysed (Table 2). Nest identity from which the isolated colony came from was in any case the best explanatory variable since it explained the largest amount of variation in microbial activity of *Enterococcus* colonies isolated from uropygial gland secretions of hoopoes (Table 2).

**Table 2:** Univariate results of the percentage of variance explained (R^2^) by each predictor variable in the three PCA axes.

	PCA 1			PCA 2			PCA 3		
	R^2^	F	P	R^2^	F	P	R^2^	F	P
**Nest**	54.72	36.31	<0.0001	63.84	45.93	<0.0001	43.47	11.95	<0.0001
**Individual**	40.89	22.11	<0.0001	21.27	12.47	<0.0001	37.23	8.33	<0.0001
**RAPD group**	52.89	18.83	<0.0001	12.18	4.7	<0.0001	30.71	4.53	<0.0001

## Discussion

Most of the bacterial colonies isolated from the hoopoe’s uropigial gland demonstrated antimicrobial activity against different bacterial strains. Actually, in all the nests we found at least one bird whose bacterial isolates had the capacity of inhibiting at least one indicator strain. Moreover, at the individual level, all but three individuals hosted at least one colony demonstrating antimicrobial activity. Rates of antimicrobial activity of isolated colonies at the level of individual hoopoes is very similar to that detected for uropygial secretions (90% in [16]), which further suggests a link between the presence of enterococci and the antimicrobial activity of the uropygial gland secretion of hoopoes, probably due to the secretion of bacteriocins [15], [16], although other non-peptide compounds may also be involved [13].

Different bacteria isolated from the same individual hoopoe UGS were more frequently included as members of the same genetic (RAPD) group than those from different individuals. Moreover, bacteria isolates from different individuals from the same nest included members of the same RAPD group more often than expected by random. This result shows inter-individual and inter-nests differences in the genetic profile of symbiotic bacteria living in the UGS of hoopoes.

Importantly, we have found that isolates differed considerably in their ability to inhibit the indicator bacteria strains assayed. This variation was explained by the genetic profile of the colony, the identity of the individual hoopoe and the nest from which the colony came from. This result indicates that benefits (in terms of antimicrobial protection) for hoopoe nestlings would vary depending on the identity of symbionts. Given that individuals from the same nests shared the genetic profile of symbiotic bacteria, they would share also the antimicrobial activity conferred by these symbiotic bacteria. Interestingly, this environmental effect would be the result of indirect genetic effects (*sensu* [21]) if the symbiotic bacteria detected in the UGS of individuals within the same nest were associated with particular bias in nest selection by adults, or with vertical transmission of bacteria from brooding females to nestlings.

The antimicrobial activity of bacteria from different RAPD groups was quite variable both in power (size of inhibition halo) and spectrum (between 0 and 8 indicator strains affected). This variation in the antimicrobial potential of symbiotic bacteria assembled to different individuals implies a gradation in the protection that symbionts can confer to hosts against other microbes. Therefore, those communities including bacteria with different inhibition spectrum will defend their hosts from a wider range of bacteria, which will be of selective advantage.

The antagonistic activity of symbionts may be due to the production of different chemicals such as volatiles [13] and bacteriocins [15], both of them products of the bacterial metabolism, which varied among individuals and therefore are subjected to selection. Interestingly, the inhibition spectrum found in the present study coincides with that of the bacteriocin MRR10 isolated from hoopoes [15] and thus it is possible that part of the antimicrobial activity detected here were mediated by bacteriocin production. The genus *Enterococcus* is particularly prolific in bacteriocin production [14], and their antagonistic spectrum (i.e., number of bacterial strains against which they are active) is highly variable among producer strains [22]. Most bacteriocins have been described to out-compete closely related species [14]. However, we found that our isolates were effective also against other non-related bacterial groups, which reveal a wide inhibition spectrum. The broad spectrum of isolates from UGS, therefore, suggests that bacteriocins from symbionts may protect hoopoes from some potentially pathogenic bacteria (e.g. some strains of the genera *Staphylococcus*, *Listeria*, and *Bacillus*) growing in the nests and nestling’s plumage. Nevertheless, chemical composition of the culture medium (BHA) used in our experiments, but also laboratory environmental conditions, differs from these of the uropygial secretion and gland of hoopoes. Several factors such as growing medium, pH, temperature, glucose concentration, or availability of other nutrients are known to affect bacteriocin activity [23] and, thus, further analyses should consider the inhibitory activity against pathogenic strains trying to recreate the natural conditions and confronting the symbiotic bacteria with their natural competitors. In this sense, we have demonstrated that the bacteriocin producer strain *E. faecalis* MRR 10 can prevent feather degradation by *B. licheniformis* [17].

To sum up, we have here detected a relationship between antimicrobial activity of enterococci isolates from hoopoés uropygial glands and the genetic profile of the bacteria strain, the individual and the nest identity from where the isolate came from. These results suggest that benefits that hoopoes could receive from symbionts would vary among individuals and nests. Differences between symbionts in their effectiveness as antimicrobial tools may have an indirect genetic component and, consequently, be subject of natural selection.

## Materials and Methods

### Study Area, Bacterial Sampling and Isolation

The field work was performed during the 2003, 2005 and 2006 breeding seasons in the “Hoya de Guadix” (SE Spain), where hoopoes breed in nest boxes (see [24] for more detailed description of the study area). All necessary permits were obtained from the Andalucía Regional Government for the field studies and sampling.

The UGS was extracted from nestlings 19–21 days after hatching of the first egg, and from incubating females. Samples were collected with a micro-pipette by introducing the tip within the papilla of the gland where the secretion accumulates. Before extraction, the circlet feathers of the gland were separated, and the gland was slightly washed with ethanol to prevent contaminations from external bacteria. Secretion from each nestling was stored in a sterile microfuge tube at 4°C until laboratory analyses, which were performed within the next 48 hours. No damage was ever inflicted to individuals when the secretion was extracted.

We isolated symbiotic bacteria by spreading 5 µl of a 1∶5 dilution (into sterile distilled water) of each UGS onto a plate with the general culture medium Tryptic Soy Agar (Scharlau, Barcelona, Spain). Plates were incubated aerobically for 24 h at 37°C and, afterwards, five randomly selected colonies from each plate were transferred to tubes containing Brain Heart Infusion (BHI, Scharlau, Barcelona, Spain) dissolved in sodium phosphate buffer (0.1 M, pH 7) plus 0.8% agar. After 18 h, 500 µl of the dilution (the culture of the isolated strain in BHI) was kept in 500 µl of sterile 70% glycerol at −80°C. To analyse the samples, each isolated strain in glycerol was spread onto Brain Heart Agar (BHA, Scharlau, Barcelona, Spain) plates, and incubated for 24 h at 37°C. We incubated 286 bacterial isolates from 58 individual hoopoes (2 females and 56 nestlings) belonging to 24 nests. We used a single sample of uropygial gland secretion per individual hoopoe.

### Study of Antimicrobial Activity of the Different Isolates

The antimicrobial activity of the isolates was tested according to the double layer technique [25]. Each colony was replicated by spotting onto BHA, and incubated 24 h at 37°C. After growing, plates with 30–35 replicated colonies were covered with 6 ml of BHI dissolved in sodium phosphate buffer (0.1 M, pH 7) plus 0.8% agar, inoculated with 100 µl of a 24 h culture of the indicator strains, and then incubated 24 h at 37°C.

Antimicrobial activity tests were performed against a total of 11 bacterial typified strains from a wide range of bacterial taxa: from the Spanish Type Culture Collection (CECT), *Escherichia coli* CECT774, *Listeria inocua* CECT4030, *Listeria monocytogenes* CECT4032, *Micrococcus luteus* CECT241, *Salmonella choleraesuis* CECT443 and *Staphylococcus aureus* CECT240; *Bacillus licheniformis* D13, *Enterococcus faecium* UJA34, *Enterococcus faecalis* S-47 and *Klebsiella* sp. from our own laboratory collection, and *Lactococcus lactis* LM 2301 [26]. The antimicrobial activity was revealed by the appearance of clear growth-inhibition halos around the spots of isolates. The intensity of the activity was marked as 0 (no halo), 1 (a small ring around the colony, width <1 mm), 2 (ring width = 1–2 mm), 3 (ring width = 3–4 mm), and 4 (ring width >4 mm.).

### Typification of the Genetic Profile (RAPD) of Isolates

For genetic analyses, 1 ml of a 24 h BHI culture of each isolate was centrifuged and cells were frozen at −20°C until DNA extraction. Bacterial genomic DNA was extracted using the Modified Salting-Out Procedure procedure [27]. To type the isolates, the method of Randomly Amplified Polymorphic DNA (RAPD) was used [28]. DNA amplification was made in a Biorad Gene Cycler (Bio-Rad) using the primer M13 (5′-GAGGGTGGCGGTTCT-3′) at a concentration of 1 µM in the reaction mixture. Amplification reactions were as follows: a denaturing step of 94°C 1 min, 35 cycles of 94°C 1 min, 40°C 20 seconds (0.6°C/s ramp) and 72°C 80 seconds, finished by an elongation step at 72°C 5 min. Amplified DNA fragments were separated electrophoretically (at 30 volts) during 16 h on 1.2% agarose gels, using the 1-Kbp Ladder (Biotools, Madrid, Spain) as molecular size standard, stained in ethidium bromide (0.5 µg/ml), and visualized under UV light.

Gels were analysed with the Fingerprinting II Informatix Software 2000 (Bio-Rad, Hercules, CA) to cluster the isolates. Similarity coefficients between fingerprints were calculated with the Pearson correlation coefficient. A dendogram was constructed with the Unweighted Pair Group Method with Arithmetic mean (UPGMA) algorithm. RAPD and cluster analyses were repeated with randomly selected samples, and we found that the same isolates were always grouped in a similarity above 80%, allowing us to establish the identity level at a 80% of similarity, so samples clustered with similarities >80% were considered to belong to the same RAPD group. Moreover, in 12 groups established by RAPD, more than one isolate was identified by 16S rDNA sequencing. All the sequences belonging to the same RAPD group were identified as the same species, which support our approach of grouping the isolates with a similarity above 80% in their genetic profile.

The use of RAPD-PCR analysis as well as the way of defining RAPD groups is a useful tool, and reproducibility is achieved under the same controlled conditions [29].

### Identification of Bacterial Isolates

We performed a preliminary identification of the isolates based on a few phenotypic traits for the colonies isolated from samples of 2003 and 2005 breeding seasons (N = 45). All of them were assigned to the genus *Enterococcus* according to the cell morphology and Gram-staining reaction, catalase reaction**,** growth and aesculin hydrolysis on bile-aesculin agar (40% bile v/v), growth at alkaline pH and in the presence of 6.5% NaCl [30], [31].

In addition, in 60 randomly selected isolates (14 of 2003 and 2005, i.e. 31.11% of the phenotypically determined isolates, and also 46 isolates of 2006) belonging to 40 different RAPD groups, the gen 16S rRNA was sequenced [32]. A 700-bp fragment of the 16S rRNA gene, which included variable regions V1 to V4, was amplified for representative strains of each RAPD genotype. The PCR was carried out using a 50 µl (total volume) mixture containing 5 µl of 10× *Taq* reaction buffer, 10 µl of 5× *Taq* Enhancer, 1.5 mM magnesium diacetate, each dNTP at a concentration of 400 µM, 0.4 µM primer WO1 (5′-AGAGTTTGATC[AC]TGGCTC-3′), 0.4 µM primer WO12 (5′-TACGCATTTCACC[GT]CTACA-3′), 1 U of Eppendorf Master *Taq* polymerase, and 1 µl of template DNA. The amplification program consisted of an initial denaturing step of 94°C for 4 min, followed by amplification using 30 cycles of 30 s at 94°C, 30 s at 50°C, and 60 s at 72°C and then a final extension at 72°C for 2 min. PCR products were purified with a Perfectprep gel cleanup kit (Eppendorf, Hamburg, Germany). The sequence of the 16S rRNA was determined by using CEQ 2000 dye terminator cycle sequencing with a Quick Start kit (Beckman Coulter, California, USA) according to the manufacturer's instructions. The resultant sequence was analyzed with a CEQ DNA analysis system (version 4.0). The overlapping sequences obtained were merged using the Lasergene program, version 5.05 (DNASTAR, Inc., Madison, WI). A search for homology of the DNA sequence was made using the BLAST algorithm [33] available at the National Center for Biotechnology Information (NCBI, USA).

### Statistical Analyses

We compared the probability of detecting bacterial isolates of the same RAPD group within the same individual with that of isolates of different RAPD groups being randomly distributed among individuals. Only individuals with more than one colony (N = 57), and RAPD groups that appeared in more than one individual (N = 38) were used. Furthermore, to explore whether a target bacterial isolate that was detected in a target nestling were more frequently detected in individuals of the same nests than what it should be expected by random, we used bacterial RAPD profiles that were detected in more than one nest (N = 19). For each of those isolates, we compared the probability of detecting bacterial colonies belonged to the RAPD group in individuals within the same nests with that of colonies of different RAPD groups being randomly distributed among sampled individuals from different nests (N = 52 nestlings). Frequency distributions of these probabilities did not differ significantly from normality (Kolmogorov-Smirnov, all p-values >0.05) and, thus, pair-wise t-tests for all statistical comparisons were used.

Antimicrobial activity against *E. coli*, *S. choleraesuis* and *Klebsiella* sp. were not detected and thus were not used for statistical analyses. Values of antimicrobial activity against the remaining eight indicator strains were included in a Principal Component Analysis (PCA) to reduce the number of dependent variables and assure statistical independence among them. PCA factors were rotated (varimax normalized), and their significance established by cross-validation. Finally, antagonistic activity was summarized in 3 Principal Components that explained 85.57% of the variation in the PCA. PC1 explained 61.29% of the variation and was positively related to the antagonistic activity against *L. innocua* (factor loading = 0.86), *L. monocytogenes* (factor loading = 0.89), *E. faecalis* (factor loading = 0.90) and *E. faecium* (factor loading = 0.75). The second component (PC2) explained 16.18% of variance and was positively related with *B. licheniformis* (factor loading = 0.80), *S. aureus* (factor loading = 0.87) and *M. luteus* (factor loading = 0.80) antagonistic activities. Finally, PC3, that explained 8.08% of the variation reflected the antibacterial activity against *L. lactis* (factor loading = 0.89). The three resulting PCA appropriately caught variation of the 8 variables since estimated powers for each of them varied between 0.69 and 0.96 [34].

A multivariate General Linear Model (GLM) was performed, in which the factors obtained in the PCA were the dependent variables; nest identity, individual identity (nested within nest identity), and identity of the RAPD group were the predictor variables. All the analyses were performed with Statistica 6 software (StatSoft 2001).
